# Association Between Serum Triglyceride Levels and the Severity of Acute Pancreatitis: A Retrospective Study

**DOI:** 10.7759/cureus.78053

**Published:** 2025-01-27

**Authors:** Mohid Bin Tahir, Arham Bin Tahir, Fatima Meer, Iftikhar Hussain, Md Shamimur Rahman, Muhammad Rawal Saeed, Ubaid Ur Rehman, Rana Shahzaib Ali, Abdul Eizad Asif, Ali Ahmed, Tayyab Mumtaz Khan

**Affiliations:** 1 Internal Medicine, Jinnah Hospital, Lahore, PAK; 2 Emergency Medicine, Beaumont Hospital, Dublin, IRL; 3 Emergency Medicine, Swansea Bay Health Board, Swansea, GBR; 4 Internal Medicine, Rawalpindi Medical University, Rawalpindi, PAK; 5 Internal Medicine, University Hospital of Coventry and Warwickshire, Coventry, GBR; 6 Neurosurgery, National Hospital for Neurology and Neurosurgery, London, GBR; 7 Trauma and Orthopedics, Rawalpindi Medical University/Benazir Bhutto Hospital, Rawalpindi, PAK; 8 Neurosurgery, Rawalpindi Medical University/District Headquarters Teaching Hospital, Rawalpindi, PAK; 9 Internal Medicine, Sheikh Zayed Medical College and Hospital, Rahim Yar Khan, PAK; 10 Internal Medicine, Shalamar Medical and Dental College, Lahore, PAK; 11 General Surgery, Avicenna Medical and Dental College, Lahore, PAK; 12 Orthopedic Surgery, Rawalpindi Medical University, Rawalpindi, PAK

**Keywords:** acute, association, pancreatitis, ranson, serum, severity, triglyceride

## Abstract

Background

Acute pancreatitis (AP) is a potentially fatal condition with a poor prognosis if it escalates to its severe form. The pathophysiology of AP has been associated with serum triglyceride (TG) levels. However, the relationship between serum triglyceride (TG) levels and AP severity remains poorly understood. Therefore, this study aimed to investigate the correlation between serum TG levels and the severity of AP.

Methods

This retrospective study was conducted at Benazir Bhutto Hospital (BBH) in Rawalpindi, Pakistan, from January 2023 to January 2024, among 210 patients with AP. Data was gathered on a self-devised proforma from medical records. The Ranson criteria score was utilized to assess the severity of AP. Patients were divided into two groups: patients with non-severe AP and patients with severe AP on the basis of their Ranson criteria score. Data analysis was done in the Statistical Package for Social Sciences (SPSS) version 25 (IBM Corp., Armonk, NY) through the chi-squared test, independent t-test, Pearson's correlation, and a simple linear regression analysis. The p-value of less than 0.05 was set as significant.

Results

Of the 210 patients, 135 (64.28%) had non-severe AP, while 75 (35.72%) had severe AP. Values of the variables including gender, age, white blood cell (WBC) count, serum aspartate transaminase (AST) levels, serum blood glucose levels, serum lactate dehydrogenase (LDH) levels, Ranson score, serum amylase levels, serum lipase levels, body mass index, serum TG levels, and length of hospital stay were significantly (p < 0.05) higher among the patients with severe AP. Pearson's correlation indicated that serum TG levels were positively and significantly associated with the Ranson scores (coefficient {r} = 0.80; p < 0.003). Moreover, linear regression analysis confirmed serum TG level as a significant predictor of AP severity, with a beta coefficient (β) of 3.21 and a 95% confidence interval (CI) of 1.82-4.72 (p < 0.002). The frequency of clinical outcomes such as pancreatic necrosis, intensive care unit (ICU) admission, and mortality was significantly higher among the patients with elevated serum TG levels.

Conclusions

In the current study, elevated serum TG level was found to be an independent predictor of the increased severity of AP, as suggested by higher Ranson scores and adverse clinical outcomes among the patients with raised serum TG levels. This current study supports the use of serum TG level as a reliable tool for the prompt identification of high-risk AP patients, facilitating timely interventions and improved outcomes.

## Introduction

Acute pancreatitis (AP) is a sudden and severe inflammation of the pancreas, causing abdominal pain, nausea, vomiting, and potentially life-threatening complications [1,2]. The symptoms of AP can range from mild to severe and may include abdominal pain, nausea, vomiting, fever, and jaundice [1,2]. Globally, the incidence of AP varies, with an estimated 34 cases per 100,000 people per year. AP is a common cause of hospitalization, with an estimated 210,000 cases per year in the United States alone [3]. In Pakistan, the incidence of AP is estimated to be around 13-45 cases per 100,000 people per year [4].

The pancreas plays a vital role in the digestion and metabolism of lipids and glucose regulation; any disruption to its function can have severe consequences. If left untreated, AP can lead to severe complications, pancreatic necrosis, pancreatic pseudocyst, pancreatic fistula, pancreatic ascites, splenic vein thrombosis, recurrent acute pancreatitis, chronic pancreatitis, intensive care unit (ICU) admission, and even mortality [5,6]. The mortality of acute pancreatitis ranges from 4% to 25%. AP exhibits two peaks of severity. Initially, there is a high risk of death within the first two weeks of illness, accounting for up to 50% of all deaths related to AP. Later, a second peak of severity emerges due to infected necrosis-induced sepsis, posing an additional threat to patients [7]. However, mortality related to AP depends upon two factors including the timely identification of the severity of AP and the adequate treatment of AP. Treatment modalities for AP vary depending on the severity of the disease. Mild cases of AP can be managed conservatively with fluid resuscitation, pain management, and nutritional support. Severe cases of AP may require intensive care unit (ICU) admission, mechanical ventilation, and surgical intervention [1,8].

Several scoring systems have been used to assess the severity of AP, including the Ranson criteria, the Acute Physiology and Chronic Health Evaluation (APACHE) II score, and the Bedside Index of Severity in Acute Pancreatitis (BISAP) score. Although these scoring systems help clinicians predict the severity of AP and guide treatment decisions, these are complex to deal with as multiple investigations are required during their score calculation [2,9].

In recent years, serum triglyceride (TG) level has emerged as a reliable tool for predicting acute pancreatitis (AP) severity and guiding clinical decision-making [10]. Serum TG levels have gained attention due to their simplicity, cost-effectiveness, availability of test results within a few hours, crucial role in lipid metabolism, and potential association with the inflammatory mechanisms that produce AP. Increased serum TG levels may aggravate pancreatic injury via lipotoxicity and inflammatory substances. There is no inherent toxicity of triglycerides to the pancreas. Triglycerides are broken down by the pancreatic lipase into free fatty acids, which cause lipotoxicity. The severity of injury to the pancreas depends mainly on inflammatory response and lipotoxicity-induced damage to the pancreas [11-13].

The relationship between serum TG levels and AP severity is not well established as some studies demonstrated that serum TG levels are significantly associated with AP severity [14-19]. However, other studies presented no correlation between elevated serum TG levels and severity and clinical outcomes of AP [20-22]. These differences in the findings of different studies about the association between serum TG levels and AP severity emphasize how crucial it is to carry out new research to completely comprehend this connection.

Furthermore, there is also a significant gap in research in Pakistan on the correlation between serum TG levels and AP severity. Therefore, this study aims to address this research gap by investigating the correlation of serum TG levels with AP severity and clinical outcomes in Pakistani patients with AP. By doing so, it seeks to contribute to the development of serum TG level's role in the early identification and management of AP. Ultimately, this research aims to facilitate timely treatment, prevent fatal complications, and improve outcomes for AP patients.

## Materials and methods

Study design and study population

This retrospective cohort study was carried out at Benazir Bhutto Hospital (BBH) in Rawalpindi, Pakistan, over one year, from January 2023 to January 2024. A total of 210 patients with acute pancreatitis managed in BBH were enrolled in the study. A 15% prevalence of acute pancreatitis from a study by Alam et al., with a 5% margin of error and a 95% confidence interval (CI), was used to calculate this sample size. The convenient sampling technique was used for the present study [1]. The validity of the study was ensured by applying strict inclusion and exclusion criteria.

Inclusion and exclusion criteria

Patients diagnosed with acute pancreatitis of any gender with an age of 18 years or above and complete medical records, including all investigation reports that were required for the assessment of the severity of AP and serum triglyceride (TG) levels, were included in the study. On the other hand, patients with an age of less than 18 years and a history of previous acute pancreatitis, biliopancreatic surgery or pancreatic trauma, antibiotic treatment within the last 24 hours, initial treatment at another hospital, steroid use within the last three months, chemotherapy or radiation therapy within the previous six weeks, alcohol intake, lipid disorders, blood disorders, malnourishment, and chronic inflammatory diseases were not included in the current study. We excluded patients with the abovementioned conditions as these could impact the findings of the study by increasing or decreasing the severity of AP and serum triglyceride levels among the patients with AP.

Primary outcomes and secondary outcomes

Investigating the relationship between serum TGs and the severity of acute pancreatitis (AP), as determined by the Ranson criteria, was the primary objective of this study. Three secondary outcomes/objectives were also sought by the study in addition to this main goal. First, serum TG levels were compared in two different patient groups: those with severe and non-severe AP. Second, to ascertain serum TG level's potential use as a prognostic marker, the study also evaluated its predictive ability for the evolution of AP severity. The third one was about the comparison of outcomes of AP between the patients with elevated serum TG levels and patients with normal serum TG levels.

Acute pancreatitis and its severity measurement

Acute pancreatitis is the acute inflammation of the pancreas that is diagnosed by the presence of any two of the following conditions: acute abdominal pain and an increase in serum lipase (U/L) or serum amylase (U/L) of three times the upper limit of normal [1]. The severity of AP was assessed using the Ranson criteria score. The Ranson criteria utilize 11 parameters to assess the severity of acute pancreatitis (AP). Upon admission, five parameters are evaluated: age over 55 years, white blood cell (WBC) count exceeding 16,000 cells/mm³, blood glucose levels of 200 mg/dL (11 mmol/L) or higher, serum aspartate aminotransferase (AST) levels of 250 IU/L or higher, and serum lactate dehydrogenase (LDH) levels above 350 IU/L. An additional six parameters are checked at 48 hours, including serum calcium levels below 8.0 mg/dL (2.0 mmol/L), a hematocrit fall of 10% or more, partial pressure of oxygen (PaO_2_) levels of 60 mmHg or lower, a blood urea nitrogen (BUN) increase of 5 mg/dL or more despite intravenous fluid hydration, a base deficit of 4 mEq/L or higher, and the sequestration of fluids exceeding 6 L. The severity of AP is then determined by the total score, with scores of 2 or lower indicating non-severe AP and scores of 3 or higher indicating severe AP. In the current study, the Ranson criteria assessment was based on five parameters on admission: age of >55 years, WBC count of >16,000 cells/mm³, blood glucose of ≥200 mg/dL (11 mmol/L), serum AST of ≥250 IU/L, and serum LDH of >350 IU/L at admission. The reason for selecting the Ranson criteria was primarily due to its widespread use and acceptance in our institution [2].

Serum triglyceride levels

Patients with serum TG levels of <150 mg/dL were considered to have normal serum TG levels, while patients with serum TG levels of ≥150 mg/dL were marked to have elevated serum TG levels [10].

Sample collection for the required investigations

In compliance with hospital regulations that follow guidelines established by the College of American Pathologists (CAP), registered nurses took blood samples from every patient. In order to measure the Ranson score at admission, these samples were examined to identify different blood parameters.

Data collection

For the present study, data was collected from medical records on a self-structured questionnaire that had three distinct parts. The first part was regarding demographic information (age and gender), past medical history, and physical examination findings. The second part was about the laboratory test reports. The third part used data from the first two parts to calculate the Ranson criteria score.

Data analysis

The Statistical Package for Social Sciences (SPSS) version 25 (IBM Corp., Armonk, NY) was used for statistical analysis. Frequencies and percentages were used to describe qualitative data, whereas mean ± standard deviation was used to summarize quantitative data. The two study groups' numerical and nominal variables were compared using the independent t-test and chi-squared tests, respectively. The association between Ranson scores and the serum TG levels was investigated using Pearson's correlation analysis. Additionally, the predictive value of serum TG level for Ranson scores was evaluated using a linear regression model. P-values below 0.05 were regarded as statistically significant.

Ethics

The Ethical Review Board (ERB) of Benazir Bhutto Hospital in Rawalpindi, Pakistan, granted ethical permission for this study (ERB number: BBH.ERB.283.269).

## Results

Among the 210 patients, non-severe acute pancreatitis was identified in 135 (64.28%) patients, while severe acute pancreatitis was found in 75 (35.72%) patients. The frequencies of patients with normal serum TG levels and elevated TG levels were 155 (73.81%) and 55 (26.19%), respectively.

Table 1 shows the study population's demographic and clinical features. It has also demonstrated significant differences between the two study groups (non-severe AP group and severe AP group) in several primary parameters that included gender, age, WBC count, serum AST levels, serum blood glucose levels, serum LDH levels, Ranson score, serum amylase levels, serum lipase levels, body mass index, serum TG levels, and length of hospital stay, with p < 0.05.

**Table 1:** Demographic and clinical characteristics of the study population along with independent t-test and chi-squared test analysis The sub-rows of the second row in the second, third, and fourth columns show the number and percentages of the variable gender, while the rest of the rows of the second, third, and fourth columns show means along with the standard deviation of different study variables. In the second to the last column, test statistic values with the sign + are of the Independent t-test, while test statistic values with the sign * are of the chi-squared test. Similarly, in the last column, p-values with the sign + are of the independent t-tests, while p-values with the sign * are of the chi-squared test. Note: One of the secondary outcomes of the present study is the comparison of the serum TG levels between two different patient groups such as patients with severe and non-severe AP. P < 0.05, statistically significant; t-value, a test statistic for independent t-test; X² value, a test statistic for chi-squared test AST, aspartate transaminase; LDH, lactate dehydrogenase; BMI, body mass index; N, study population size; n, sample size for each group or category; %, percentage

Variables for Patients With Acute Pancreatitis (AP), N = 210		Expression of Variables	Severity of Acute Pancreatitis		Chi-Squared (χ²) Test/Independent T-Test	
					Test Statistics	
			Non-severe AP Group, n = 135 (64.28%)	Severe AP Group, n = 75 (35.72%)	χ² Value for Chi-Squared Test/T-Value for Independent T-Test	P-Values
Gender	Male	148 (70.48%)	100 (74.07%)	47 (62.66%)	3.84^*^	0.04^*^
	Female	62 (29.52%)	35 (25.93%)	28 (37.34%)		
Age (Years)		61.94 ± 16.12	56.49 ± 12.62	64.22 ± 12.32	4.32^+^	0.004^+^
White Blood Cell Count (Cells/mm^3^)		14.54 ± 6.67	14.41 ± 4.30	18.74 ± 5.93	4.15^+^	0.002^+^
Serum AST Level (IU/L)		245.54 ± 134.48	253.32 ± 44.10	286.44 ± 154.06	2.53^+^	0.001^+^
Blood Glucose Level (mg/dL)		240.70 ± 60.36	150.34 ± 36.99	250.46 ± 52.76	6.21^+^	0.003^+^
Serum LDH Level (IU/L)		520.46 ± 120.22	390.17 ± 70.99	569.77 ± 130.09	7.34^+^	0.002^+^
Ranson Score		3.09 ± 1.34	1.40 ± 0.47	3.80 ± 1.18	13.42^+^	0.001^+^
Serum Amylase Level (U/L)		410.12 ± 120.48	250.45 ± 130.10	509.33 ± 180.32	5.67^+^	0.002^+^
Serum Lipase Level (U/L)		459.32 ± 150.15	340.72 ± 140.08	550.06 ± 170.41	5.19^+^	0.003^+^
BMI (kg/m²)		26.47 ± 4.24	25.19 ± 3.04	29.67 ± 5.40	4.53^+^	0.005^+^
Serum Triglyceride (TG) Level (mg/dL)		190.47 ± 75.24	170.19 ± 40.02	264.45 ± 90.40	4.32^+^	0.004^+^
Length of Hospital Stay (Days)		7.47 ± 3.24	5.19 ± 1.02	9 ± 3.40	7.45^+^	0.002^+^

Table 2 reveals a statistically significant positive relationship between serum TG levels and the severity of AP as determined by Pearson's correlation analysis. This positive correlation shows that as serum TG level rises, Ranson scores also tend to increase, indicating a direct relationship between serum TG level and AP severity.

**Table 2:** Correlation between serum TG levels and the severity of acute pancreatitis in the study population In the second and third columns, mean values with standard deviation of study variables are given. T-value: A test statistic for independent t-test. Note: Investigating the relationship between serum TGs and the severity of acute pancreatitis (AP), as determined by Ranson criteria, is the primary objective of this study

Variables, N = 210	Severity of Acute Pancreatitis		Independent T-Test		Pearson's Correlation	
			Test Statistics		Test Statistics	
	Non-severe AP Group	Severe AP Group	T-Value	P-Value	Correlation Coefficient (r)	P-Value
Ranson Score	1.40 ± 0.47	3.80 ± 1.18	13.42	0.001	0.80	0.003
Serum Triglyceride (TG) Levels	170.19 ± 40.02	264.45 ± 90.40	4.32	0.004		

Table 3 demonstrates that the simple linear regression model was exceptionally fit (R² = 0.80; p < 0.0001), showing a statistically significant positive association between serum TG levels and Ranson scores. A positive beta coefficient indicates that a higher serum TG level was correlated with the increased Ranson score, indicating greater AP severity.

**Table 3:** Determination of the predictive value of serum TG levels for the severity of acute pancreatitis via simple linear regression model Note: Assessing the serum TG level's potential use as a prognostic marker among patients with AP is one of the important objectives of the study CI: confidence interval

Variable	Test Statistics for Simple Linear Regression Model				
	Unstandardized Regression Coefficient (β)	95% CI	P-Value	R^2^ Value	P-Value of F-Test
Serum Triglyceride (TG) Levels	3.21	1.82-4.72	0.002	0.80 (80.00%)	0.000

Table 4 summarizes the clinical outcomes among the patients with AP. This table also shows that the frequency of pancreatic necrosis, intensive care unit admission, and mortality was higher among the patients of AP with elevated serum TG levels as compared to those who had normal serum TG levels, and variations in the frequency of these parameters between the two groups were statistically significant, with p < 0.05.

**Table 4:** Clinical outcomes according to serum TG levels in the study population In the last three rows of the second and third columns, frequency along with percentages of different clinical outcomes is shown. X² value: A test statistic for the chi-squared test. Note: Evaluating the various clinical outcomes' percentages among the patients is also one of the significant objectives of the study TG, triglyceride; ICU, intensive care unit

Variables	Distribution of Study Population Based on the Serum TG Levels		Chi-Squared Test	
			Test Statistics	
Outcomes	Normal TG Group, n = 155 (73.80%)	Elevated TG Group, n = 55 (26.20%)	χ² Value	P-Value
Pancreatic Necrosis	2 (1.29%)	6 (10.90%)	10.33	0.001
ICU Admission	8 (5.16%)	12 (21.81%)	9.32	0.002
Mortality	1 (0.65%)	4 (7.27%)	6.34	0.001

## Discussion

AP is a life-threatening condition, and it leads to a significant global healthcare burden. The management of AP is supportive in case of early identification. A quicker and more effective approach increases the likelihood of survival and prevention of complications in the patients of AP [1,4,6]. Recently, various studies have shown that serum triglyceride (TG) level is a valuable predictor of acute pancreatitis (AP) severity and clinical outcomes of AP [10,11]. The simplicity, cost-effectiveness, and rapid availability of TG test results make them particularly useful. Moreover, TG plays a crucial role in lipid metabolism and is linked to inflammatory mechanisms that contribute to AP. Elevated TG levels can exacerbate pancreatic damage through lipotoxicity and the release of inflammatory substances [12,13]. In the present study, we have determined valuable data regarding the correlation of serum TG levels with the severity of AP. Moreover, we have also evaluated the differences in the means of primary study parameters among two study groups such as patients with non-severe AP and severe AP. These parameters include age, gender, WBC count, serum AST levels, serum blood glucose levels, serum LDH levels, Ranson score, serum amylase levels, serum lipase levels, body mass index, serum TG levels, and length of hospital stay.

In addition to the determination of the correlation between AP severity and serum TG levels, frequencies of clinical outcomes including pancreatic necrosis, ICU admission, and mortality were also compared between the patients with normal serum TG levels and patients with elevated serum TG levels. Elevated serum triglyceride (TG) levels contribute to the severity of acute pancreatitis through a multifaceted mechanism. Initially, high TG levels lead to the formation of free fatty acids, which activate pancreatic inflammatory cells and release pro-inflammatory cytokines. These cytokines exacerbate pancreatic injury, promoting the activation of digestive enzymes and the release of toxic mediators. Furthermore, TG-rich lipoproteins accumulate in pancreatic tissue, causing oxidative stress and mitochondrial dysfunction. Impaired lipid metabolism also leads to the accumulation of toxic lipid intermediates, such as lysophosphatidylcholine, which further worsen pancreatic damage. The resulting inflammatory cascade amplifies pancreatic injury, leading to the increased severity of acute pancreatitis. An increased serum TG levels indicate an imbalance between these immune responses, predicting poor outcomes as the unchecked inflammatory response causes tissue damage, organ dysfunction, and increased mortality [11,12].

In the present study population, 135 (64.28%) patients had non-severe AP, while 75 (35.72%) patients had severe AP. A Turkish study has reported almost similar results about the two categories of AP as in this study; 67 (64.43%) patients had non-severe AP, while 37 (35.57%) patients had severe AP [2]. Conversely, in a South Korean study, in comparison to the current study, a lower frequency of severe AP and a higher frequency of non-severe AP have been noted [23]. The present study population's AP severity differs from that of studies carried out in various countries for different reasons, such as the etiology, presentation of patients, and environment.

Demographic characteristics had a great influence on the severity of AP as the frequency of patients with raised severity of AP was greater among male patients. The rising shift in the severity of AP was also observed with the rise in age among the study population. Different investigations have also noted the similar role of demographics in patients with AP and endorsed these findings of the present study [4,7].

Among the two study groups, patients with non-severe AP and severe AP, statistically significant differences in the means of primary study variables including, age, WBC count, serum AST levels, serum blood glucose levels, serum LDH levels, Ranson score, serum amylase levels, serum lipase levels, body mass index, serum TG levels, and length of hospital stay were noted (p < 0.05). In the literature, many studies that used the Ranson scoring system for the assessment of AP severity and to correlate it with serum TG levels have supported these findings of the present research about the key factors of the Ranson criteria score, Ranson score, and serum TG levels [10,13].

In the present study, serum TG level was positively and significantly correlated with AP severity as patients with elevated severity of AP had raised serum TG levels. The principal finding of this study was congruent with the many previous studies that were conducted in various parts of the globe. Another study from the literature has also shown the function of serum TG level as a significant and reliable predictor of AP severity [14]. A study from China has also mentioned that the association between raised serum TG level and the increased severity of AP and high serum TG level anticipates poor prognosis among patients with AP [15]. Similarly, a meta-analysis has consistent findings regarding the significant association between serum TG level and the severity of AP as in this current study [16]. A Spanish study has also presented comparable observations regarding serum TG level and AP severity. It has demonstrated that with the elevation in serum TG level, the severity of AP increases [17]. Another international multicenter cohort study, in which data was collected from different countries including the United States, Europe, Latin America, and India, has also noted that patients with higher serum TG levels had more severe AP in comparison to the patients with lower serum TG levels [18]. Another study has also found a variation in serum TG levels in the different categories of patients with different severities of AP [19]. The findings of this current study support the use of serum TG level as an accurate and effective biomarker for determining the severity of AP, and they are identical to previous studies.

Regarding clinical outcomes, our study revealed that patients with elevated serum triglyceride levels (≥150 mg/dL) had higher rates of pancreatic necrosis (10.90% versus 1.29%, p = 0.001), ICU admission (21.81% versus 5.16%, p = 0.002), and mortality (7.27% versus 0.65%, p = 0.001) as compared to those with normal triglyceride levels. Specifically, our findings showed that patients with hypertriglyceridemia were more likely to experience severe disease, resulting in increased mortality, requiring ICU admission, and developing pancreatic necrosis. These results are consistent with previous studies, which have also reported poorer outcomes among patients with elevated serum triglyceride levels compared to those with normal levels [10,14,24].

This research has significant implications for the determination of the severity of AP by using serum TG levels, and it has also laid the groundwork for future prospective investigations, which can further explore the implications of our findings for the diagnosis, management, and treatment of acute pancreatitis. Identifying elevated serum TG levels as a predictor of disease severity enables clinicians to stratify patients according to risk and tailor treatment strategies accordingly. The early recognition of high-risk patients allows for prompt intervention, including aggressive fluid resuscitation, nutritional support, and monitoring for complications. Moreover, this association suggests that lipid-lowering therapies, such as dietary modifications and fibrate use, may have a role in reducing the severity of acute pancreatitis during future episodes of AP [11,12]. Further studies are needed to explore the therapeutic potential of targeting lipid metabolism in acute pancreatitis and to develop evidence-based guidelines for managing hypertriglyceridemia in this context.

Our study provides valuable insights into the prognostic value of serum TG levels in acute pancreatitis (AP) while we compare it with the gold standard Ranson score for AP. However, it also has some limitations, including a relatively small sample size, a single-setting population, a retrospective study design, no control of confounder factor age, and no calculation of the specific serum TG level that could predict poor outcomes among the patients with AP. These limitations may restrict the generalizability of our findings. To address these limitations, future studies with larger sample sizes, mixed populations, different study designs including prospective and experimental studies, control of confounder factor, and calculation of the specific serum TG level that could predict poor clinical outcomes in AP patients, are necessary to validate our results and further establish the prognostic value of serum TG levels in AP.

## Conclusions

This study has demonstrated a significant positive association between serum triglyceride levels and the severity of acute pancreatitis. Higher serum TG levels were consistently correlated with the increased AP severity. A simple linear regression model confirmed this positive association between serum TG levels and AP severity. Similarly, patients with raised TG levels had a higher percentage of adverse outcomes that included pancreatic necrosis, ICU admission, and mortality. These findings suggest that serum TG level along with other prognostic factors can be used as a reliable predictor of AP severity for the crucial and timely management of AP. The determination of the serum TG levels among AP patients is recommended to facilitate timely interventions and improve patient outcomes. Clinicians are advised to consider serum TG levels alongside other diagnostic tools for comprehensive AP severity assessment. Incorporating serum TG level assessment into clinical practice may lead to improved outcomes and reduced mortality among AP patients, by providing additional prognostic information and the early stratification of the patients with acute pancreatitis.

## Appendices

Table 5 shows the research questionnaire.

**Table 5:** Research questionnaire for the association between serum triglyceride levels and the severity of acute pancreatitis

Sections	Research Questions	Options: Write/Tick the Option	
Section A (History and Physical Examination)			
1. What Was the Age of the Patient? (Years)			
2. What Was the Gender of the Patient?		Male	Female
3. What Were the Presenting Complaints of the Patient?			
4. Previous History of Acute Pancreatitis/Hepatobiliary Surgery?		Yes	No
5. Past Treatment History?		Yes (If Yes, Then Describe the Details)	No
6. Presence of Any Chronic Disease?		Yes (If Yes, Then Describe the Details)	No
7. What Were the Physical Examination Findings of the Patient? (Vitals, General, and Systemic)			
8. What Was the Body Mass Index of the Patient? (Weight {kg} / {Height {m}}^2^)			
9. What Was the Length of Hospital Stay?			
10. What Was/Were the Clinical Outcome/s of Patients?			
Section B (Laboratory Reports)			
1. Serum Amylase or Lipase Level (U/L)			
2. White Blood Cell Count (Cells/mm^3^)			
3. Serum Aspartate Aminotransferase (IU/L)			
4. Blood Glucose Level (mg/dL)			
5. Serum Lactate Dehydrogenase (IU/L)			
6. Serum Triglyceride Level (mg/dL)			
Section C (Ranson Score Calculation and Patient Division Into Non-severe Acute Pancreatitis and Severe Acute Pancreatitis)			
1. Ranson Score			
2. Severity of Acute Pancreatitis	Non-severe Acute Pancreatitis Group	Severe Acute Pancreatitis Group	
